# Study of the bone tissue structural-functional state in children in the East of Ukraine

**DOI:** 10.1186/1546-0096-12-S1-P299

**Published:** 2014-09-17

**Authors:** Tatiana Frolova, Olga Okhapkina, Irina Tereshchenkova, Iryna Siniaieva

**Affiliations:** 1 pediatrics, Kharkov national medical university, Kharkov, Ukraine

## Introduction

The problem of osteopenia and osteoporosis in children turned from a rare pediatric problem to a frequent pathology which is difficult to diagnose. This problem is especially urgent for the present-day in Ukraine because the level and quality of therapeutic and diagnostic care as well as nutrition quality are influenced by the transformation of the society. The critical years for building bone mass are during childhood and adolescence. This is when a new bone is formed more quickly than the old bone is removed, causing bones to become larger and denser.

## Objectives

The 1126 children aged 9-17 residing in the East of Ukraine.

## Methods

The ultrasound densitometry.

## Results

According to the findings of population study we worked out special nomograms for evaluation of structural-functional state of the bone tissue in children population in Ukraine for the use by medical practitioners. Active accumulation of the bone mass is observed between the age of 11-12, its increase equals 7-8% per year. The periods of intensive growth and active bone mass accumulation occur at the same time. The incidence of osteopenic disorders in the bone tissue structural-functional state in children aged 9-16 in a large industrial area is (20.5±1.1)% and changes with the age, gender, residence from (14.6±2.3)% to (30.3±4.0)%. In the children, the incidence of stage 1 osteopenia is (9.3±1.1)%, stage 2 – (7.1±0.8)%, stage 3 – (4.1±0.5)%. Thus, the most frequent variant of osteopenia is stage 1 disturbance (43.3% of the cases). 20.0% of cases are marked osteopenic disorders (stage 3). Age- and gender-related differences in the values of osteopenia prevalence in children are characterized by prevail of its frequency in the young group (9-12 years) over the older group, which is more pronounced in girls ((24.8±1.8)% and (18.2±2.0)%, respectively, p<0.05), first of all due to the larger frequency of stage 3 osteopenia ((6.4±1.2)% and (3.5±0.8)%, respectively, p<0.05). In girls from rural regions, osteopenia incidence was (16.7±1.9)%. In the younger age group, stage 1 and stage 3 osteopenia were more prevalent (9.4±3.3)% and (6.4±1.7)%, whereas in the older age group the correlation between the prevalence of these variants is smaller. In urban girls osteopenia prevalence was (26.1±2.5)%, in the younger group, stage 1 osteopenia was more prevalent (12.1±3.8)% and stage 3 osteopenia was less prevalent (2.1±1.7)%, while in the older group correlation of these variant of osteopenia is smaller. In rural boys, the prevalence of osteopenia was (15.7±2.1)%. In the younger age group, the lowest incidence and, respectively, stage 3 incidence (2.8±1.1)% were noted. In children aged 9-12, osteopenia prevalence was (18.0±2.2)%; 51.1% of cases were stage 2-3 disease. In children aged 13-16, stage 2-3 osteopenia made 54.8%. Osteopenia prevalence in urban children was revealed to be (24.3±2.7)%. In children aged 9-12 this made (27.3±2.8)%, 83.6% of cases being stage 1-2 osteopenia. The number of stage 1-2 cases reached 85.6% in the children aged 13-16, which suggests accumulation of osteopenic disorders in the teen-agers.

## Conclusion

The necessity of implementation of nomograms for evaluation of the bone tissue structural-functional state in children in regions with different ecological and medical and social characteristics was proved.

## Disclosure of interest

None declared.

